# Lipoprotein Receptor-Related Protein 4 Antibody Positivity in the Youngest Patient in the Caucasus Region: A Case Report

**DOI:** 10.7759/cureus.68961

**Published:** 2024-09-08

**Authors:** Javid Sardarzada, Banu Anlar

**Affiliations:** 1 Pediatric Neurology, Leyla Medical Center, Baku, AZE; 2 Pediatric Neurology, Güven Hospital, Ankara, TUR

**Keywords:** autoantibody, double seronegative (dsn), juvenile myasthenia gravis (jmg), low-density lipoprotein receptor-related protein 4 (lrp4) antibodies, myasthenia gravis (mg)

## Abstract

Juvenile myasthenia gravis is a rare disorder where antibodies targeting the acetylcholine receptor or, less frequently, muscle-specific kinase can be detected in the serum while about half of the patients can be seronegative. A pediatric patient with ocular myasthenia is presented whose serum was negative for acetylcholine receptor and muscle-specific kinase antibodies but tested positive for low-density lipoprotein receptor-related protein 4 antibodies. A favourable clinical response was observed to medical treatment with pyridostigmine and prednisolone, as expected in isolated ocular juvenile myasthenia gravis. This case exemplifies the very rare association of juvenile myasthenia gravis with low-density lipoprotein receptor-related protein 4 positivity, reported in only a few cases so far. The specificity of the antibody and the efficiency of medical treatment emphasize the importance of clinical suspicion and appropriate serological testing in juvenile myasthenia gravis in the absence of acetylcholine receptor and muscle-specific kinase antibodies.

## Introduction

Myasthenia gravis (MG) is the most prevalent disorder of the neuromuscular junction (NMJ) where antibody-induced dysfunction results in muscle weakness and fatigue [1]. Pathogenic antibodies target acetylcholine receptors (AChR) in the majority of MG patients; less commonly, antibodies against muscle-specific kinase (MuSK), agrin, and low-density lipoprotein receptor-related protein 4 (LRP4) are identified [1]. LRP4 is a transmembrane protein which forms complexes with other NMJ proteins MuSK, agrin, and ColQ and plays a crucial role in the clustering and function of the AChR [2]. Antibodies targeting LRP4 can be detected in 18.7% of MG cases who are seronegative for anti-AChR and anti-MuSK (double seronegative (dSN)) and also in some seropositive for anti-AChR (7.5%) or anti-MuSK (15%) [3].

Childhood-onset MG or juvenile myasthenia gravis (JMG) is relatively uncommon, constituting 10-15% of all MG cases [4]. To our knowledge, anti-LRP4-positive JMG has mainly been reported from Asian countries and in only one 17-year-old from Europe so far [5]. We present a child with isolated ocular myasthenia seropositive for LRP4 antibodies, one of the youngest in the literature.

## Case presentation

This 11-year-old girl was referred to our department by an ophthalmologist for unilateral ptosis and diplopia. Her complaints had started 20 days ago and showed diurnal fluctuation.

The patient is the first child of healthy, non-consanguineous parents. Her past medical and developmental history were unremarkable, and her school performance is satisfactory. Her 10-year-old brother is healthy.

Neurological examination revealed ptosis of the eyelid covering approximately half of the pupil on the left and limitation of extraocular movements in both eyes. In addition, lateral gaze in the left eye and medial gaze in the right eye were restricted. Other physical and neurological findings were normal.

**Figure 1:**
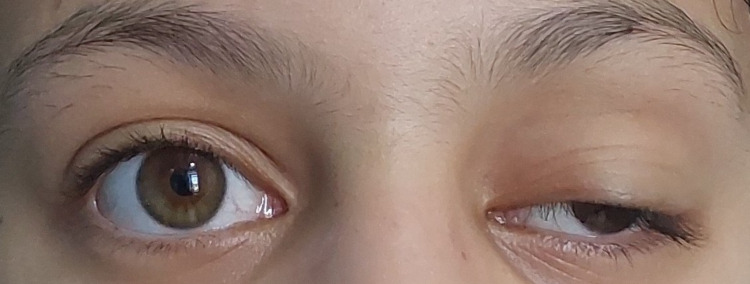
Before oral pyridostigmine

**Figure 2:**
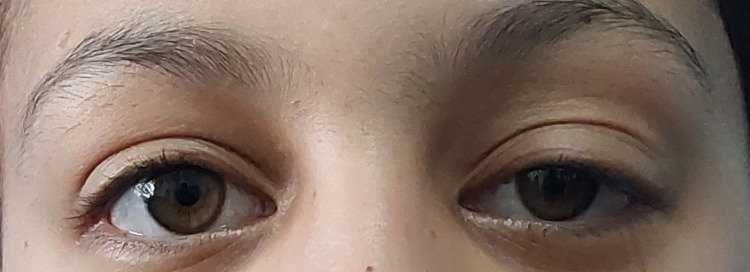
Improvement of ptosis 30 minutes after oral pyridostigmine

The ice pack test and an oral dose of pyridostigmine 60 mg resulted in a dramatic improvement of the eye movements and ptosis (Figure 1 and Figure 2). Serum antibodies against AChR, MuSK, thyroglobulin, and thyroperoxidase tested negative, but the anti-LRP4 IgG index was positive at 3.5 (negative: <2.1) by enzyme-linked immunosorbent assay (ELISA).

**Figure 3:**
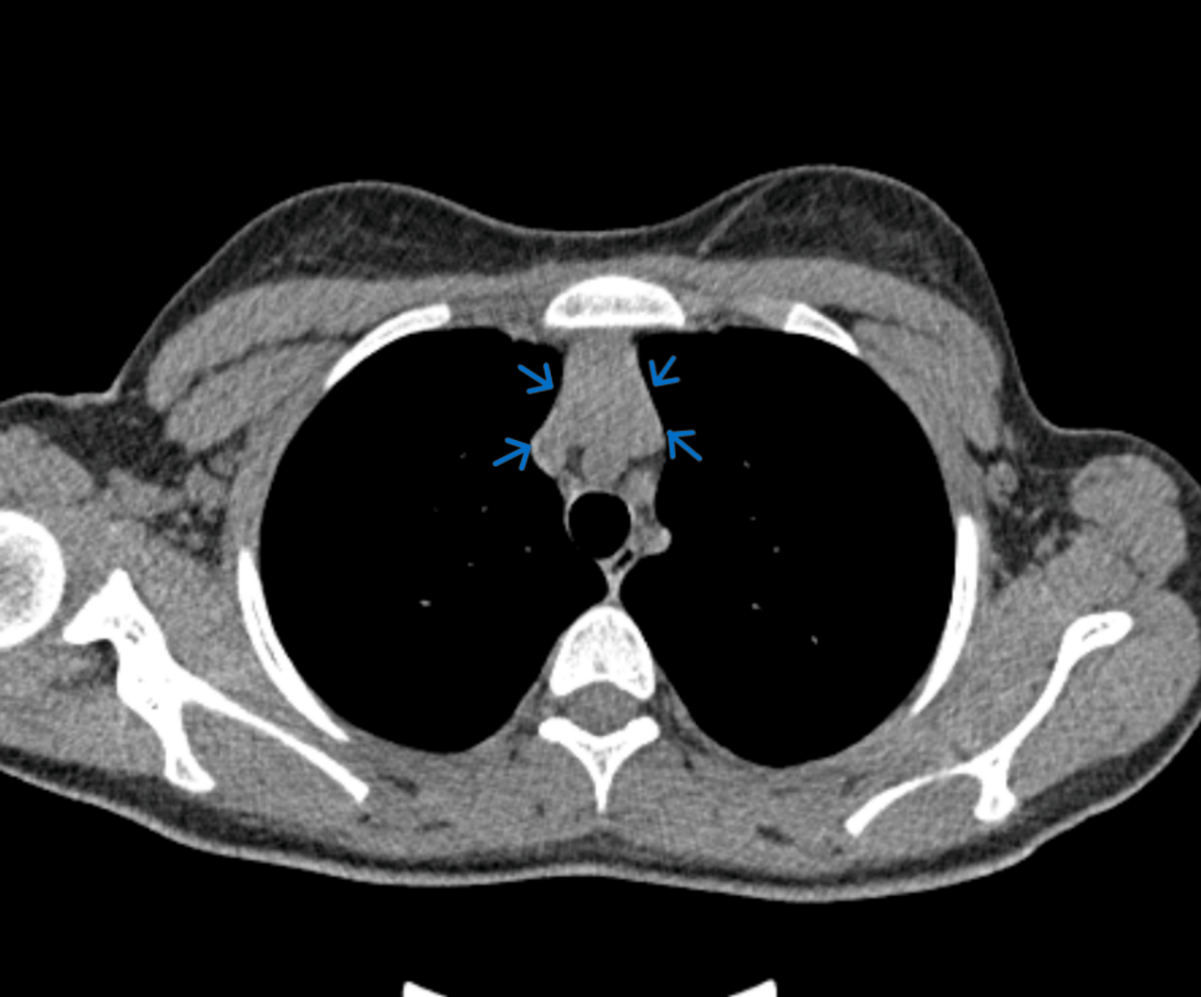
The chest CT scan in thymic hyperplasia CT: computed tomography

Cranial magnetic resonance imaging (MRI) and electromyography (EMG) with repetitive stimulation of limb muscles yielded normal results. The chest computed tomography (CT) scan revealed thymic hyperplasia (Figure 3).

The patient was initiated on treatment with pyridostigmine 60 mg PO three doses per day. Symptoms had improved at one-month follow-up: mild ptosis and minimal restriction of the eye movements were observed and no diplopia was reported. Immunosuppressive therapy or thymectomy was not considered at that stage in view of the favourable response to treatment. However, right-sided ptosis developed three months after the diagnosis. Increasing the daily dose of pyridostigmine to four times 60 mg did not show any effect. Oral prednisolone was started and her symptoms subsided, resulting in clinical remission.

## Discussion

Juvenile MG is a rare disorder. Anti-LRP4-positive MG is also uncommon, constituting 0.7% of all MG cases [5]. The average age of onset of anti-LRP4-positive dSN MG is 33.4 years for women and 41.9 years for men [3]. Therefore, anti-LRP4 seropositivity in JMG is a very rare association, reported mostly from East Asia so far [5]. Li et al. collected 455 cases of dSN MG, of whom 13 were anti-LRP4 positive and these patients were <14 years old [5]. Higuchi et al. identified only six anti-LRP4-positive patients among 272 with dSN-MG and none in the pediatric age group [6]. An adolescent from Germany is the only case of anti-LRP4-positive JMG reported from Europe [7].

In adults, anti-LRP4-positive MG tends to manifest with moderate generalized weakness rather than isolated ocular involvement; however, symptomatology has been reported in small series only [3,7]. In JMG, the rates of ocular vs. generalized vary between series. The former is common in JMG series from China, reported as high as 93.6% [8]. In the anti-LRP4-positive JMG series, the largest also from China, extraocular movements are reported as the most common initial presentation as well (10/13, 77%) [5].

Among clinical investigations, repetitive nerve stimulation (RNS) rarely shows abnormal findings in LRP4 MG: almost all patients in the anti-LRP4-positive and anti-LRP4-anti-MuSK double-positive groups had negative RNS and overall lower decrement values compared to anti-AChR or anti-MuSK antibody-positive groups [9]. EMG yielded normal results in our patient but was performed on limb muscles which were clinically not involved.

The role of the thymus in the pathogenesis of anti-LRP4 MG and the need for a chest CT are unclear. Aoki et al. reported a case with thymoma [10]. None of the LRP4 MG patients in Zisimopoulou et al.'s series had thymoma, but 31% had thymic hyperplasia [4], as did ours. Thymectomy is generally not advised in dSN MG as this group is less likely to respond to this treatment although certain anti-LRP4 MG patients have been reported to improve after thymectomy [2,10]. As cholinesterase inhibitors, corticosteroids, and, in some patients, azathioprine are expected to induce favourable responses, the treatment of anti-LRP4 MG is generally conservative [3,6,8].

## Conclusions

LRP4 antibodies should be considered in patients presenting with isolated ocular JMG who are seronegative for AChR and MuSK. Although very rare in the pediatric population, the detection of LRP4 antibodies can expedite the diagnosis of JMG, allow the prompt initiation of treatment, and avoid unnecessary investigations such as cranial MRI.
